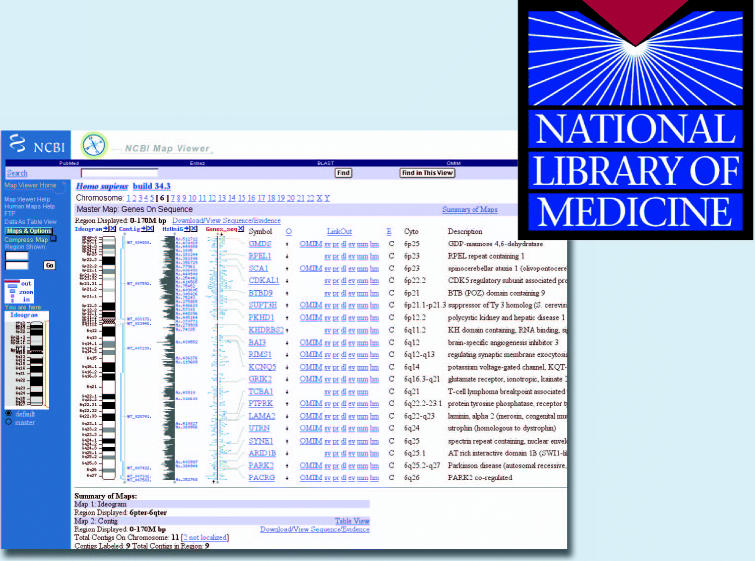# TXGnet: National Center for Biotechnology Information

**DOI:** 10.1289/ehp.112-1277128

**Published:** 2004-08

**Authors:** Erin E. Dooley

The National Center for Biotechnology Information (NCBI) of the National Library of Medicine was created as a means of developing new information technologies to help advance the field of molecular biology. Composed of a group of scientists from disciplines including mathematics, research medicine, and structural biology, the center’s programs and activities are centered on both basic and applied research in computational molecular biology. Since the center’s inception in 1988, NCBI scientists have developed important novel algorithms and research approaches in the fields of computational biology and gene sequencing, among others. Today, researchers and others can find an impressive array of “omics” resources and tools collected into one central resource on the NCBI website at **http://www.ncbi.nlm.nih.gov/**.

General information on the center’s work can be found within the top center section of the homepage as well as by clicking the About NCBI link on the left side of the homepage. A large portion of the NCBI web-site is devoted to the number of free, publicly accessible databases and software programs that the center hosts. Included among the databases on offer are GenBank, the Online Mendelian Inheritance in Man, the Cancer Genome Anatomy Project, and numerous organism-specific genome databases. The site classifies these databases as literature search databases, molecular databases, or genomic biology databases to make finding them easier; GenBank, NIH’s annotated genetic sequence database, stands under its own heading. The Molecular Databases page—which is based on Entrez, the integrated search and retrieval system developed by the NCBI—has more than 25 additional database classifications such as nucleotide, protein, and expression. Entrez can also be quickly accessed through a pull-down menu at the top of the homepage.

The Genomic Biology section of the site provides a brief overview of this relatively new area of science. Visitors to this section will find a wealth of human genome resources, including a map viewer that can be used to browse the human genome. By selecting the Human Genome Resources page, users can access PDF background documents on the databases available on the site, two of which provide instructions on how to use the map viewer to explore genomes.

The main portion of this Genomic Biology area is divided into sections on genes and human health and contains links to Online Mendelian Inheritance in Man, RefSeq, dbSNP, and Gene Database. Visitors can also access BLAST for comparing genomic sequences and gene products, and a centralized registry of genomic clones, end sequences, mapping data, and distributor information. Other tools are available as well, and are classified under Maps and Markers, Transcribed Sequences, Cytogenetics, and Comparative Genomics.

The right-hand toolbar for the Genomic Biology section contains resources for 20 specific organisms. Selecting any one of these organisms takes the user to a guide outlining the many different search tools and other resources offered by the NCBI and other groups. These resources include gene maps, sequences, and annotation projects.

Educators and others using the site can access an online guide to GenBank and NCBI resources through the Education link on the homepage. Also on the Education page are tutorials for BLAST, Entrez, and other tools available on the site, as well as access to NCBI newsletters and map viewer exercises. Also within this section is an online science primer developed by the NCBI as a way to familiarize nonspecialists with terms and concepts such as “genome mapping,” “expressed sequence tags,” and “phylogenetics.”

## Figures and Tables

**Figure:**